# Spatial and temporal multiplet analysis for identification of dominant fluid migration path at The Geysers geothermal field, California

**DOI:** 10.1038/s41598-021-03267-y

**Published:** 2021-12-13

**Authors:** M. Staszek, Ł. Rudziński, G. Kwiatek

**Affiliations:** 1 Institute of Geophysics, Polish Academy of Sciences, Warsaw, Poland; 2 Helmholtz-Centre Potsdam, GFZ German Research Centre for Geosciences, Section 4.2: Geomechanics and Scientific Drilling, Potsdam, Germany

**Keywords:** Natural hazards, Solid Earth sciences

## Abstract

Multiplet analysis is based on the identification of seismic events with very similar waveforms which are used then to enhance seismological analysis e.g. by precise relocation of sources. In underground fluid injection conditions, it is a tool frequently used for imaging of subsurface fracture system. We identify over 150 repeatedly activated seismic sources within seismicity cluster induced by fluid injection in NW part of The Geysers geothermal field (California). Majority of multiple events (ME) occur along N–S oriented planar structure which we interpret as a fault plane. Remaining ME are distributed along structures interpreted as fractures, forming together a system of interconnected cracks enabling fluid migration. Temporal analysis reveals that during periods of relatively low fluid injection the proportion of ME to non-multiple events is higher than during periods of high injection. Moreover, ME which occur within the fault differ in activity rate and source properties from ME designating the fractures and non-multiple events. In this study we utilize observed differences between ME occurring within various structures and non-multiple events to describe hydraulic conditions within the reservoir. We show that spatial and temporal analysis of multiplets can be used for identification and characterization of dominant fluid migration paths.

## Introduction

Multiplets, i.e. seismic events with high level of waveform similarity, are successfully used for identification of subsurface fractures and fracture systems in underground fluid injection conditions. Groups of very similar seismic waveforms are used as an input into high-accuracy relocation methods, such as double-difference relocation method^1–3^ or collapsing method^4^. As a result, an image of fracture network and local tectonic structures can be obtained. Fracture network mapping using multiplet analysis has been performed in many previous studies, e.g. by Lees at Coso geothermal field^5^, Moriya et al*.* at Soultz-sous-Forêts geothermal field^6^, Mukushira et al*.* at Basel enhanced geothermal system^7^ or Got et al*.* beneath Kilauea Volcano^2^.

In tectonic settings multiplets, also called repeaters, are generally interpreted as an indicator of aseismic creep^8^. The energy is then repeatedly released on specific fault patches, whereas the remaining part of the fault plane is slipping slowly without generating any earthquakes. However, in underground fluid injection environments multiplets can be generated also by simple repeating activation of the same fault plane due to variations in injection activity and resulting pore pressure fluctuations. Such events have been described e.g. during Deep Heat Mining project in Basel, Switzerland by Goertz-Allmann and Wiemer^9^. The biggest number of event repetitions was observed close to the injection well where pore pressure was the highest. On the other hand, aseismic genesis of multiplets in fluid injection settings has been described e.g. at Soultz-sous-Forêts geothermal field in France^10–12^. Finally, an occurrence of multiplets can be explained by an activation of separate similarly oriented fractures, as suggested by e.g. Goertz-Allmann et al*.* in case of carbon capture and storage Illinois Basin–Decatur Project in USA^13^.

In fluid injection environments shear failure on the fault is generally agreed to be a result of pore pressure increase which leads to the decrease of effective normal stress and fault strength^14,15^. Therefore, according to Mohr–Coulomb criterion the pore pressure level needed to activate the fault depends on the initial proportion of shear stress to effective normal stress acting on this specific fault plane. At this point, the orientation of the fault plane in relation to the local stress field and following slip tendency analysis^16^ becomes an important issue. This problem was studied e.g. by Martinez-Garzόn et al*.*^17^ who showed that within Prati-9 and Prati-29 seismicity cluster from The Geysers geothermal field (California) the majority of fault planes are favorably oriented for failure and pore pressure excess needed for reactivation is < 10 MPa.

Significant variations of static stress drop (*∆σ*) within injection induced seismicity datasets have been observed and, in some cases, related to pore pressure variations^18–21^. Staszek et al*.*^20^ observed an inverse relation between *∆σ* and injection rate, whereas Goertz-Allmann et al*.*^18^ and Kwiatek et al*.*^22^ described *∆σ* increase with the distance from the injection well. The proposed physical process standing behind it is the same in both cases: a decrease of effective normal stress and fault strength due to pore pressure increase. Assuming that such relation is valid, *∆σ* can be treated as a proxy of pore pressure distribution in the reservoir. However, there are some other factors possibly influencing *∆σ* values such as: hypocentral depth (e.g.^23^), rock type or its level of damage (e.g.^24,25^). The reservoir within main fracture network or fault damage-zone is possibly more cracked and weakened than within areas activated less often. Summarizing, we expect to observe relatively low *∆σ* values within main seismogenic zones or areas of increased pore pressure level.

In this study we identify multiplets within isolated seismicity cluster induced by fluid injection at The Geysers geothermal field and use them to image underground fracture network. Moreover, we describe the dynamics of identified fractures’ activation and compare *∆σ* between groups of multiple events and single events. Finally, potential physical processes responsible for differences in seismic characteristics of identified fractures are discussed.

## Study area and data

We use the seismic and associated data from a distinct seismicity spatio-temporal cluster associated with injection activities into Prati-9 and Prati-29 wells in the northwestern part of The Geysers geothermal field^26^. The maximum horizontal stress direction in this area is N/NE and has been determined using earthquake fault plane solutions^27,28^. It is consistent with the orientation of regional geological structures^29^. Six Quaternary surface faults extending to the reservoir depth have been identified within NW part of The Geysers field. The faults are steeply dipping and perpendicular to each other, oriented NE-SW and NW–SE, and divide the Northwest Geysers into compartments^30^. The seismicity cluster connected with injection into Prati-9 and Prati-29 wells is separated from other clusters and injection wells by NE-SW oriented Caldwell Pines Fault and NW–SE oriented Squaw Creek Fault and Ridgeline Fault Zone (see Fig. 24 in^30^). Such isolated setting makes it a good material for seismic analysis due to the restricted number of factors influencing seismicity.

**Figure 1:**
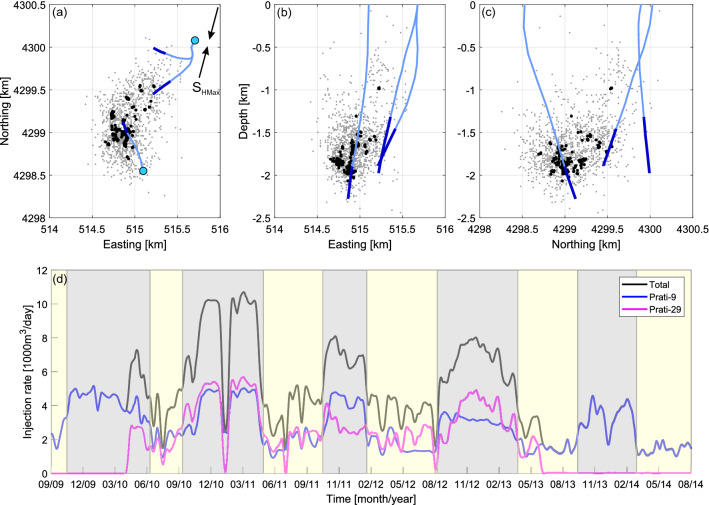
(**a**–**c**) Original localizations of 1179 catalog events (gray) and localizations of 158 multiple events after double-difference relocation (black). Trajectories of injection wells are marked with blue and their open hole sections with dark blue. (**d**) Injection rates into Prati-9 (blue), Prati-29 (magenta) and both wells (black) in time. Distinguished high and low injection rate periods are marked with gray and yellow, respectively.

The cluster has been widely studied and described in the literature (e.g.^31–33^). Prati-9 injection well was operating constantly during analyzed time period from 11/2007 till 08/2014. In the meantime, injection into Prati-29 was carried out between 04/2010 and 06/2013. Injection into both wells has seasonal character with peak injection rates occurring during winter months^30^. For the purpose of our analysis, we distinguished time periods of high and low summed injection rates into both wells (Fig. 1).

In the analysis we use seismic data registered by 31 three component 4.5 Hz geophones deployed on the surface, sampling with the frequency of 500 Hz, and operated by Lawrence-Berkeley National Laboratory (Supplementary Fig. S1). The waveform data was downloaded from Northern California Earthquake Data Center website^34^ according to seismic catalog elaborated by Kwiatek et al*.*^31^ for this region^26^. The catalog consisted of 1539 manually picked events in *M*_W_ range 1.3–3.2 (with *M*^C^_W_ = 1.4). Magnitudes were recalculated from NCEDC catalog according to the formula $M_{{\text{W}}} = 0.9 \cdot M_{{\text{D}}} + 0.47$^35^. Original localizations were taken from NCEDC website. The cross-correlation analysis was performed for entire dataset, however, in the analyses only events which occurred after 21/09/2009 were used. The reason for this choice is station exchange over the entire network which took place between 09/2009 and 01/2010. After 21/09/2009 the total number of catalog events was 1179 (Fig. 1). We used static stress drops calculated for the subset of 328 events using the spectral ratio technique^26,31^ with uncertainties estimated by Staszek et al*.*^20^.

## Results

### Identification of structures: spatial distribution of ME

**Figure 2:**
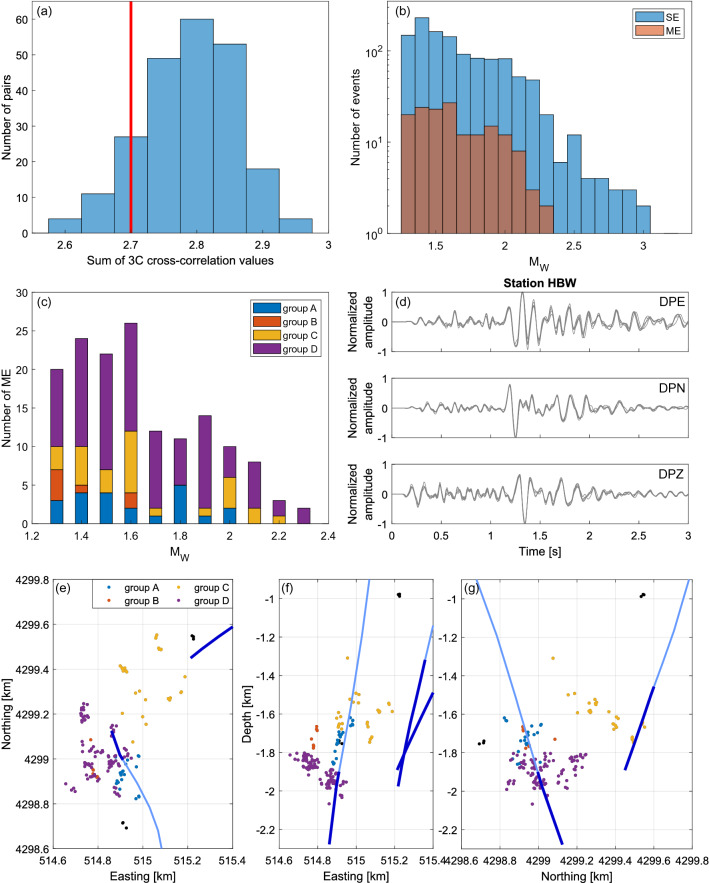
(**a**) Median three component cross-correlation values on all stations for all event pairs within identified multiplets, (**b**) moment magnitudes of all ME and SE, (**c**) moment magnitudes of ME from groups A–D, (**d**) example of the multiplet consisting of 5 events: overlaid signals after filtration to 1–15 Hz, station HBW, 3 components, (**e**–**g**) relocated ME and distinguished ME groups (A–D).

Within entire 1539-event catalog 202 events have been identified as multiplets and used in relocation procedure. However, all further analyses were performed using multiple events which occurred after 21/09/2009 in order to ensure good completeness of repeating sequences. Among 1179 events included in the catalog after 21/09/2009, 158 have been classified as ‘multiple events’ (ME). This number includes: 42 multiplets (23 triplets, 12 groups of 4 events, 4 groups of 5 events and 3 groups of 6 events) and three additional events belonging to the multiplets which started before 21/09/2009. Remaining 1021 events have been classified as ‘single events’ (SE). Detailed criteria of ME and SE classification are described in the Methods section. The histogram of 3C cross-correlation for all event pairs within the multiplets is presented in Fig. 2. We can observe a high number of pairs with 3C signal similarity in range 2.75–2.85. The small number of pairs with similarity below 2.7 is an artefact of applied clustering method^36^. Moment magnitudes of ME fall in range from 1.3 to 2.3 (Fig. 2). An example of the multiplet consisting of 5 events is presented in Fig. 2.

The majority of ME concentrate in the proximity of Prati-9 injection well and group along NW–SE axis, consistently with the well orientation (Fig. 2). However, there is also another group of ME oriented along NE-SW axis spreading between Prati-9 and Prati-29 wells. Entire ME cluster extent is ca 750 m in depth with one triplet identified ca 330 m above. The N–S and E–W extents of ME equal ca 860 m and ca 570 m, respectively. On the basis of their spatial distribution, we divided ME into four groups: A—22 events occurring along Prati-9 well (extent of ca 240 m in depth), B—7 events parallel to Prati-9 well but shifted ca 100 m to the west, C—28 events extending between Prati-9 and Prati-29 wells (extent of ca 480 m in N–S), D—95 events oriented along the plane originating from the open-hole section of Prati-9 well (Fig. 2). The orientation of the plane best fitting to the events from group D, determined using 2D regression, is N–S (strike equals 1.6°) with the dip of 37°. The orientation of planes best fitting to events from groups A and B is also N–S (strikes A: 179.2° and B: 173.4°) but with significantly larger dips of 61.2° and 80.8°, respectively. Plane fitting to events from group C exhibits their E–W orientation (strike 268.0°) with the dip of 32.2° (Supplementary Fig. S2). The structures delineated by events from groups A and B seem to originate from the plane designated by events from group D. On the contrary, the structure designated by events from group C seem to be separated and independent. Here, we interpret structures designated by ME from groups A–C as fractures, whereas group D will be treated as an image of a potential fault plane. It is important to note that the maximum horizontal stress direction in analyzed area is NNE–SSW (Fig. 1), so the potential fault is slightly rotated in relation to the local stress field. Further in the text structures designated by ME from groups A–D will be called structures A–D, accordingly.

### Dynamics of structures’ activation: ME and SE in relation to injection rate and time

#### ME versus SE

**Figure 3:**
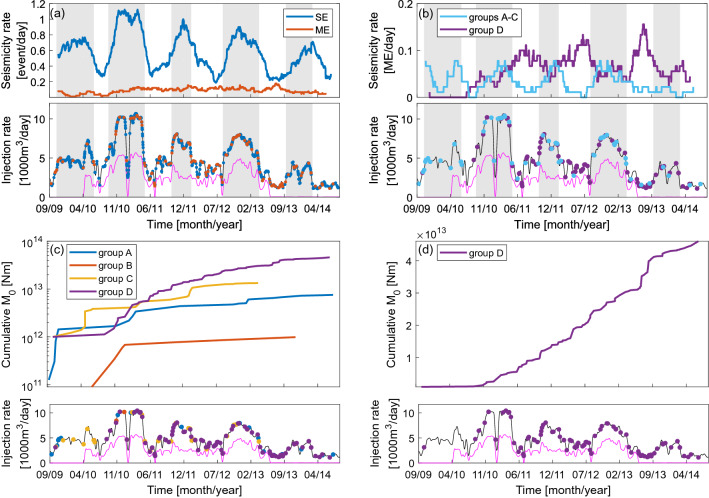
Seismicity rate of events calculated in moving window of 90 days in relation to injection rate: (**a**) SE and ME, (**b**) ME from groups A–C and D. Distinguished high injection periods are marked with gray. Cumulative seismic moment release in relation to injection rate: (**c**) ME from groups A–D in logarithmic scale, (**d**) ME from group D in linear scale.

The first aspect which needs to be described concerns the proportion of ME to SE during low and high injection rate periods. The mean ME/SE during low injection periods equals 0.24, whereas in case of high injection periods it is 0.12. This difference becomes even more interesting if we look at the seismicity rates plotted separately for ME and SE in Fig. 3. We can observe here that since the beginning of the 2nd high injection period (ca 09/2010) the overall ME rate does not depend on the injection rate (Pearson correlation coefficient *PCC* equals 0.1, *p* < 0.05) and remains constant at the level of ca 0.1 ME/day until the end of 4th injection peak. On the contrary, for SE the effect of positive short-term correlation between ongoing fluid injection and seismicity occurrence is evident (*PCC* equals 0.8, *p* < 0.05). Statistical analysis of this correlation revealed that the delay of seismicity response to injection operations is ca 2 weeks^32^. These two observations, together with the results of *Δσ* comparison, will be discussed further in terms of seismic energy budget.

#### ME: groups A–D

Temporal behavior of ME from groups A–D can be assessed using plots presented in Fig. 3. On cumulative seismic moment release curves we observe that structures A and C are activated at the very beginning of analyzed time period, whereas ME within structure B emerge since 14/06/2010. Similar situation is in the case of structure D, where only 1 event occurred before 01/09/2010. Since then, ME earthquake rate in this group increases to the mean level of ca 0.07 ME/day. Moreover, temporal fluctuations of ME earthquake rate in group D occur, which are inversely correlated with injection rate (*PCC* equals − 0.26, *p* < 0.05). These variations are observed in range from 0.02 to 0.16 ME/day. Interestingly, the ME earthquake rate peaks are becoming higher in each subsequent injection cycle. On the contrary, in case of structures A–C we can see that ME tend to occur during injection peaks. This tendency is clearly visible on cumulative seismic moment release curves, which exhibit stepwise character. Due to relatively small amount of ME within these structures and their similar temporal behavior, we plotted ME (A–C) on one rate curve. Calculated *PCC* in this case equals 0.61 (*p* < 0.05) indicating positive correlation between injection rate and ME occurrence within structures A–C. Therefore, we can summarize that the independence of ME earthquake rate on injection, described in previous section, is only apparent. In reality we can distinguish two groups of structures: (1) A–C and (2) D, responding in the opposite way to injection process. It is worth to notice that the activation of structure C, which extends between Prati-9 and Prati-29 wells, finishes simultaneously with end of injection into Prati-29. Structures A and D are being activated till the end of analyzed time period, whereas activation of structure B finishes with the last peak injection into Prati-9 well.

Total seismic moment released by ME differs significantly between the four structures (Fig. 3, Supplementary Table S1). The lowest total seismic moment is released by fracture B, designated by the smallest number of ME, and constitutes only 1.5% of total seismic moment released by all ME within structures A-D (Σ*M*_*0*_(ME)). Fractures A and C exhibit much higher total moment release on the level comparable between each other (11.1% and 19.6% of Σ*M*_*0*_(ME), respectively). The highest amount of seismic moment is released by the potential fault (structure D), where also the largest number of ME occur (67.8% of Σ*M*_*0*_(ME)). Average seismic moment release per one ME and average moment rate for each structure are presented in Supplementary Table S1. Again, structure D exhibits the highest values of both moment-related parameters. Interestingly, average seismic moment release per one ME in case of fracture C is on similar level as in case of fault D.

We observe that the first and the largest seismic moment release within structure A coincide with the first high injection period into Prati-9. Simultaneously, no influence of injection start into Prati-29 on this structure is observable. Inversely, first and also the largest seismic moment release within structure C occurs immediately after the start of injection into Prati-29. Another characteristic feature of fracture C is the occurrence of 2nd and 3rd high seismic moment releases at the end of high injection periods (differently than in case of fractures A and B). The character of seismic moment release within structure D is completely different. Since 01/09/2010 till ca 15/06/2013 the structure exhibits regular, linear growth of cumulative seismic moment (Fig. 3). After the end of injection into Prati-29 well significant increase in moment release is observed, which is connected with increased number of ME. Finally, seismic moment release rate slows down starting from September 2013.

#### ME density maps

**Figure 4:**
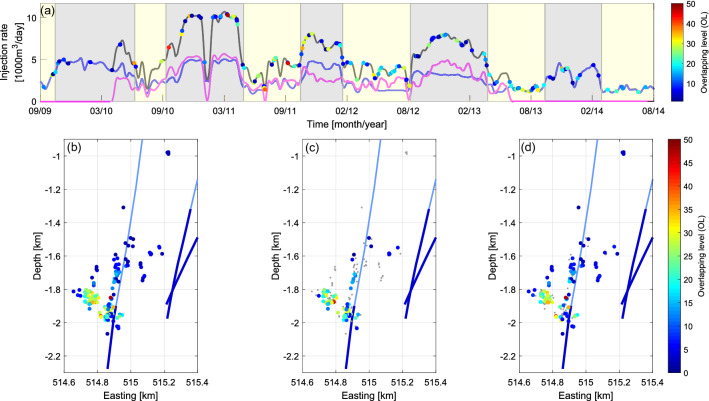
(**a**) ME occurrence in time in relation to injection rates into Prati-9 (blue), Prati-29 (magenta) and both wells (black). Colors indicate ME overlapping level. Peak and low injection rate periods are marked with gray and yellow, respectively. (**b**–**d**) Maps of ME overlapping level for: (**b**) all ME, (**c**) ME which occurred during low injection periods, (**d**) ME which occurred during high injection periods. Remaining ME are plotted as small grey dots.

**Figure 5:**
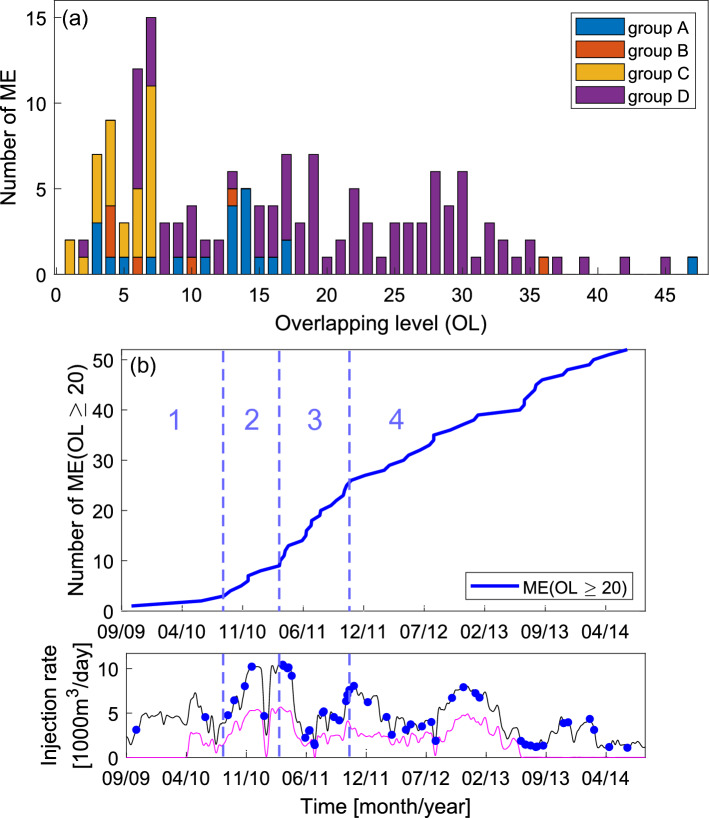
(**a**) Stacked histogram presenting OLs of ME within groups A-D. (**b**) Cumulative occurrence of ME with OL ≥ 20. Distinguished activity rate phases are marked with numbers and vertical dashed blue lines.

Figure 4 presents the results of the analysis of ME overlapping level (OL)—parameter reflecting density and overlapping of ME source areas, defined in the Methods section. In Fig. 4, plotted using ME from entire analyzed time period, we can see that ME from group D exhibit the highest OLs with the maximum and median values of 45 and 21, respectively. Lower OL values ranging mainly 3–17 are observed in case of events forming group A (median = 13; Fig. 5). ME from groups B and C present OLs almost only below 10 with both medians equal 6. It is important to note that during low injection periods mainly ME with OLs > 10 occur within groups A and D, whereas during injection peaks ME within entire OL range are observable (Fig. 4). This observation is also reflected by the median values of OL for high and low injection periods, which equal 8 and 17, respectively. According to Wilcoxon rank sum test the difference between these OL distributions is statistically significant.

An interesting observation can be made after plotting cumulative occurrence curve for ME with OL ≥ 20 (52 events), which occur within potential fault D (Fig. 5). We can see that the activity rate of these events is changing in time. Therefore, we distinguished four phases of this activity: at the beginning of the 2nd injection peak (injection rate ca 10,000 m^3^/day) activity rate accelerates from ca 0.01 ME/day to ca 0.03 ME/day. During the second part of 2nd injection peak (again ca 10,000 m^3^/day) the next acceleration to ca 0.07 ME/day is observable, which lasts till the beginning of 3rd injection peak (11/2011). After this time the occurrence of ME with OL ≥ 20 stabilize at the level of ca 0.03 ME/day. If, according to our previous considerations, we interpret structure D as a fault signature we can identify phases of its activation by analyzing highly overlapping ME rate changes.

### Stress drop comparison

Firstly, we compared *∆σ* of all ME with *∆σ* of SE. Secondly, in order to get a better understanding of physical processes provoking multiplets to occur, we compared *∆σ* of ME from fractures A-C with *∆σ* of ME from potential fault D and related them to *∆σ* of SE.

Stress drops of ME group generally in range 0.7–8.3 MPa with one event exhibiting *∆σ* of 10.2 MPa (Supplementary Fig. S3). Simultaneously, *∆σ* of SE extend generally from 0.7 to 23.8 MPa, with several events exhibiting very high *∆σ* of 28.7–58.2 MPa. The significance of *∆σ* difference between ME and SE was confirmed by Wilcoxon rank sum test at 5% significance level—among 10,000 trials only in 1016 cases (10.16%) *p*-value was above 0.05 (Supplementary Table S2). The median value of *∆σ* of ME equals 3.9 MPa, whereas in case of SE it is 4.9 MPa (Table 1). Therefore, we can confirm that in case of analyzed seismicity cluster *∆σ* of ME are significantly lower than *∆σ* of SE.

**Table 1:** Median values of *∆σ* and sample sizes in listed groups of events.

Group	Sample size	Median *∆σ* (MPa)
SE	274	4.9
ME	49	3.9
ME(C)	11	4.8
ME(D)	31	3.4
ME(A–C)	17	4.6
ME(OL < 20)	32	3.7
ME(OL ≥ 20)	17	4.3

**Figure 6:**
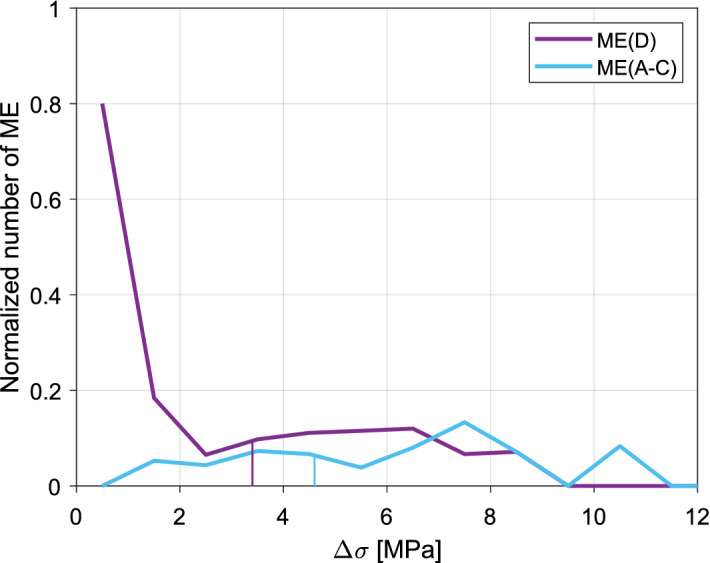
Number of ME(D) and ME(A–C) normalized to the total number of events in 1 MPa *∆σ* intervals*.* Median *∆σ* values within each group are marked with solid vertical lines.

However, if we look at this problem in more detail and compare *∆σ* of ME between structures A–D, we observe that the real group with differing *∆σ* values is the potential fault D (Fig. 6). Wilcoxon test confirms that *∆σ* of ME from structure D (ME(D)) are significantly lower than *∆σ* of ME from other structures (ME(A–C), Fig. 6) and also than *∆σ* of SE (Supplementary Table S2). Uncertainty analysis gives an unequivocal confirmation of this result in case of structure D with SE comparison. In case of interstructural *∆σ* comparison the results are less robust, possibly due to smaller sample sizes (Table 1). There is no significant difference in *∆σ* between ME with OL ≥ 20 and ME with OL < 20.

### Summary of observations

The main observations that we have made on the basis of spatial and temporal multiplet analysis of Prati-9 and Prati-29 related seismicity cluster can be summarized as follows:

1. Four structures have been distinguished within the seismicity cluster using double-difference relocation results: structures A, B and C interpreted as fractures, and structure D interpreted as a potential fault plane.
2. ME/SE ratio depends on the injection rate and is higher for low injection rate periods. Simultaneously, ME activity rate is constant over majority of analyzed time period.
3. Structures A–C and D exhibit opposite behavior in relation to injection rate. Within structure D more ME occur during low injection periods. On the contrary, within structures A–C ME tend to occur during injection peaks.
4. The highest amount of seismic moment was released by ME from structure D. The potential fault exhibits also the highest overlapping of ME. Four time periods with various activity rate have been distinguished within highly overlapping ME from structure D.
5. ME from structure D exhibit significantly lower *∆σ* than ME from other groups and SE.

## Discussion

The most important issue concerning identified structures is the difference in the occurrence and source parameters of ME between fractures A–C and fault D. Fault activation in underground fluid injection conditions is usually explained by pore pressure increase and resulting fault strength decrease^14^. Pore pressure reduces effective normal stress leading to the occurrence of seismic event. Repeating activation of fractures A–C during injection peaks is a clear reflection of described process. Multiple activation of the same structures was already proposed in the model of fluid injection induced seismicity proposed by Goertz-Allmann and Wiemer^9^. The situation is more complex in case of fault D, which is more frequently activated during low injection periods. Such observation could suggest that the fault is critically stressed and even a small pore pressure level generated during low injection periods is big enough to activate it. The instability coefficient estimated for the fault orientation of 1.6°N and dip of 37°, according to the definition of Vavryčuk et al*.*^37^ and assuming principal stress axes orientation from Martinez-Garzόn et al*.*^17^, equals 0.82. This means that according to Mohr–Coulomb criterion and assuming stress magnitudes estimated by Martinez-Garzόn et al*.*^17^, the pore pressure required for activation of fault D is ca 7 MPa. This value is comparable to the value of pore pressure estimated for periods before and after injection peaks by Martinez-Garzόn et al*.*^33^ who used data from injectivity test conducted in December 2011. Estimated pore pressure resulting from the injection only into Prati-9 well with the rate of 2725 m^3^/day equaled 2.8 MPa and 6.2 MPa at the depths of 2682 m and 3053 m, respectively. This result partially supports the thesis that the difference in structure D activation could be a result of its optimal orientation for failure. However, the values of instability coefficient for fractures A–C, estimated with the same method basing on the orientation of best fitted planes described in the “Results” section, equal 0.94, 0.86 and 0.17, respectively. Therefore, there must be also another factor promoting an activation of fault D during low injection periods over fractures A and B, which exhibit similar (B) or even higher (A) instability. And most interestingly, why there are less ME recorded on fault D during injection peaks?

For explanation of both these observations, the information about *∆σ* of multiple events can be utilized. Previous studies have shown that *∆σ* is a parameter which reflects frictional strength of the reservoir ^21^—it is lower in highly fractured areas or damage zones (e.g.^24,25^). Here, we have observed that *∆σ* of ME within structure D are significantly lower than *∆σ* of other ME and SE. Moreover, structure D generates the highest seismic moment release. Therefore, a simple explanation of faster and stronger seismic response within structure D would be its higher level of damage and lower frictional strength. This hypothesis is especially feasible if structure D is an image of a local fault with well-developed, highly permeable damage zone favoring enhanced fluid flow^38^. The presence of such local, favorably oriented for failure fault within this area has been already suggested by Martinez-Garzόn et al*.*^33^. It is important to note that ME(D) rate peaks, which coincide in time with low injection periods, are becoming higher and more concise after each subsequent injection peak (Fig. 3). On the contrary, SE rate peaks, which correlate in time with high injection periods, tend to decrease in each subsequent injection cycle (Fig. 3). Such observation could suggest that after each injection peak, damage accumulates within the reservoir fracture network that become then more prone to unload stresses on already activated discontinuities (ME) rather than activate the new ones (SE). In such reasoning, ME are treated as an indicator of damage level of the reservoir.

**Figure 7:**
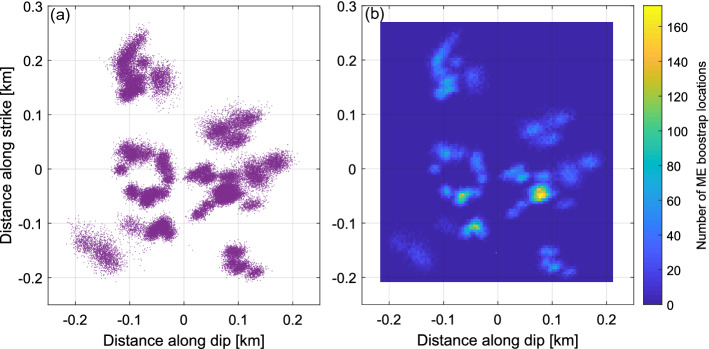
Cross section along the potential fault D plane: (**a**) hypocenters of ME and their bootstrap samples are marked with violet dots, (**b**) density map.

The physical property, which could explain decreased number of ME on fault D during high injection periods is its tendency to plastic behavior under elevated pore pressure conditions, reflected as aseismic fault movement. Multiplets, also called repeating events, are commonly interpreted in natural seismicity studies as an indicator of fault creeping (see references in^8^). Lately, many studies confirmed that in underground fluid injection conditions big amount of stress is released aseismically (e.g.^39^). In several cases multiplets were confirmed to be a signature of aseismic slip in such conditions^10,11^. The area of The Geysers geothermal field is especially prone to host aseismic deformation due to relatively high reservoir temperatures (ca 240–350 °C) and high level of rock fracturing^33^. Some previous works suggested even the possible role of aseismic deformation in case of The Geysers geothermal field^40,41^. In classical creeping fault model multiple events are generated only on locked fault patches, whereas remaining part of the fault plane is slipping aseismically^8^. Therefore, after mapping the fault plane we would expect to observe highly clustered groups of multiplets, preferably separated from each other. An image which we obtain after projecting ME(D) hypocenters, together with their bootstrap samples, on fault D plane is not far from this model (Fig. 7). We can observe that ME tend to group within 6 separate patches with one of them especially well spatially resolved. Such a fault plane image implies plastic fault behavior and occurrence of aseismic slip. This concept is consistent with observed lower stress drops of ME within this structure, where accumulated stresses are partially unloaded aseismically. Lately, the transition of fault behavior between seismic and aseismic modes has been extensively studied (e.g.^42–44^). It was proved in laboratory studies that the fault may slip in different modes during fluid injection^39,45^. Moreover, Cappa et al*.*^46^ have shown that with increasing fluid pressure friction parameters of the fault evolve from rate weakening to rate strengthening favoring its aseismic creep. Similarly, it was shown that increased pore pressure promotes slow slip and aseismic creep on areas of the subduction interface^47^. Therefore, we cannot exclude the hypothesis that fault D is behaving aseismically during high injection periods (lower ME(D) rate) and turns into rate weakening regime during low injection periods (higher ME(D) rate). An alternative hypothesis assumes continuous aseismic movement on fault D.

Finally, if we are considering aseismic movement on the fault D as a highly probably option, we need to take into account the influence of increased pore pressure and poroelastic and thermoelastic stresses on fault behavior. Thermal contraction and increased pore pressure during high injection periods may possibly lead to temporary unlocking of patches on aseismically slipping fault D (lower ME(D) rate). Then, during low injection periods, the patches are locked again and generate multiple seismic events (higher ME(D) rate) due to continuing aseismic movement of fault D. A reservoir volume increase due to fluid injection in nearby region has been confirmed by X-band interferometric measurements which revealed ca 1 cm surface uplift after start of injection operations^48^.

Finalizing our considerations concerning activation of fault D we need to remember that the structure was not fully activated until the 2nd injection peak. This observation suggests that the enhanced permeability zone either was not fully connected hydraulically with the injection well before or it developed later, after the start of injection operations. In both cases, however, the presence of preexisting local fault in this area is highly probable. Therefore, taking all above considerations into account, we interpret structure D as a local N–S oriented fault constituting well-developed enhanced permeability zone, hydraulically connected with Prati-9 injection well. We suspect high level of reservoir damage along fault D resulting in its aseismic behavior.

The following interpretation of the remaining structures A–C is proposed:

- structure A as a fracture extending along Prati-9 injection well and ending within fault zone D, influenced mainly by injection into Prati-9 well;
- structure B as a fracture parallel to fracture A and originating from fault zone D, developed during injection peak due to the migration of fluids along fault D;
- structure C as a fracture extending between Prati-29 and Prati-9 wells, oriented perpendicularly to structures A, B and D, and activated mainly by injection into Prati-29 well.

Due to observed evident influence of the end of injection into Prati-29 well on ME activity rate within most distant zone—fault D (significant increase of ME rate after the end of injection into Prati-29), we suspect that all structures A–D constitute a comprehensive, hydraulically connected crack system within analyzed seismicity cluster.

A short comment is needed regarding the observation of ME/SE ratio difference between high and low injection rate periods. We conclude from this observation that the constant part of hydraulic energy is spent on the activation of low *∆σ* ME within highly permeable and aseismically slipping fault D. Simultaneously, the excess of hydraulic energy released during injection peaks contributes to the occurrence of more violent ME within fractures A–C and SE.

## Methods

In order to identify groups of multiplets we performed cross-correlation of three component signals in time domain. We used signal windows beginning 0.1 s before *P*-wave pick and ending 3 s after it in order to quantify the similarity of *P* and *S* waveforms (*P*-wave time delays were computed later separately). Registrations from 22 stations with the best signal to noise ratio were used. Before cross-correlation signals were filtered with Butterworth filter passing frequencies 1–15 Hz (15 Hz is the maximum corner frequency estimated by Kwiatek et al*.*^31^; see^49^). For each pair of events available signals were cross-correlated accordingly to the station and component. Then, median value of cross-correlation for each component was calculated. Finally, we summed median cross-correlation values from all components obtaining a measure of 3-component signal similarity (3C cross-correlation) varying in range 0–3. The clustering of events was performed using unweighted pair group method with arithmetic mean^50,51^. The 3C cross-correlation limit equaled 2.7 (in average similarity of 0.9 per one component). Such methodology ensured that only events with high level of similarity on all 3 components were classified as multiplets. Finally, all events belonging to any group of multiplets with minimum size of 3 events were classified as ‘multiple events’ (ME) and all remaining ones as ‘single events’ (SE). It is important to note that ME which occurred before 21/09/2009 were used only for relocation purposes. All remaining analyses were performed on ME and SE which occurred after 21/09/2009 to ensure good completeness of event sequences.

All identified ME were relocated using double-difference relocation technique^3^, software version 2.1b. The method provides hypocenter locations by minimizing residuals between observed and theoretical travel-time differences at each station. Both catalog and cross-correlation data were used: catalog—*P* and *S* wave manual picks, cross-correlation—*P*-wave window starting 0.2 s before and ending 0.5 s after *P*-wave pick, with maximum lag of 0.4 s between cross-correlated events and signal frequency band of 1–40 Hz. For weighting squared cross-correlation (cross-correlation data) and station distance from the cluster centroid (catalog data) was used. Initial event locations were taken from NCEDC catalog^34^. For relocation we used data from 32 stations and utilized 1-D velocity model proposed by Eberhart-Phillips and Oppenheimer^52^. Relocation errors were estimated using bootstrap method^3^, described in detail in Supplementary Information, and presented in Supplementary Figs. S4–S9. It should be noted, that chosen relocation procedure does not account for differences in seismic velocity between hypocentral and outside rock volume. Therefore, in the calculations we assume that hypocentral rock mass is not sufficiently damaged to induce a low-velocity volume and strong velocity contrast.

Using visual inspection of relocated data, we distinguished main fracture zones within the reservoir. In order to describe the dynamics of these structures’ activation we performed the following analyses for each separate fracture: (1) temporal distribution of ME in relation to injection rate, (2) cumulative seismic moment release.

In addition, we estimated the spatial density of ME by calculating an overlapping level for each ME and plotting it on 2D maps. In order to estimate the overlapping level, we calculated parameter *η* defined by Kagan and Jackson^53^ as:

1 $$ \eta = \frac{{L_{1} + L_{2} }}{2D} $$

where *L* represents rupture length (in our case, doubled source radius) and *D* is the distance between the hypocenters. The *η* value higher than 1 implies overlapping of rupture zones. Source radii of ME were estimated with spectral fitting method described in detail by Kwiatek et al*.*^31^, using *S*-wave velocity according to 1-D model of Eberhart-Phillips and Oppenheimer^31,52^. Hypocenter distances were calculated from the relocated dataset. Using *η* estimated for every pair of ME we calculated for every event cumulative number of events overlapping with it by counting its *η* > 1 pairs. In this manner we obtained overlapping level (OL) value for every ME. The results are presented as ME density maps for entire dataset and high and low injection periods separately.

Finally, we compared static stress drops of ME from various fractures between each other and related them to *∆σ* of SE. In order to assess statistical significance of *∆σ* differences nonparametric Wilcoxon rank sum test was used at 5% significance level. The null hypothesis stated that log(*Δσ*) of events from compared groups exhibit continuous distributions with equal medians. The testing was performed for 10,000 synthetic log(*∆σ*) series where values were chosen randomly basing on log(*Δσ*) probability density functions.

## Supplementary Information

Supplementary Information.

## Data Availability

Waveform data, metadata, or data products for this study were accessed through the Northern California Earthquake Data Center (NCEDC), 10.7932/NCEDC. Raw injection data, seismic catalog and calculated source parameters are available via IS-EPOS platform of Core Service Anthropogenic Hazards: https://tcs.ah-epos.eu^26,54,55^ after registration and providing affiliation. Additional data related to this paper may be requested from the authors.
