# Supplementary material for: Spatial and temporal multiplet analysis for identification of dominant fluid migration path at The Geysers geothermal field, California
Source: Sci Rep. 2021 Dec 13;11:23908. doi: 10.1038/s41598-021-03267-y (PMC8668910; doi:10.1038/s41598-021-03267-y)
Supplement: Supplementary file 1 — Supplementary Information. [file 41598_2021_3267_MOESM1_ESM.pdf]

Supplementary Information to the manuscript:

Spatial and temporal multiplet analysis for identification of dominant fluid migration path at The Geysers geothermal field

Staszek M.[1*], Rudziński Ł.[1], and Kwiatek G.[2]

Figures

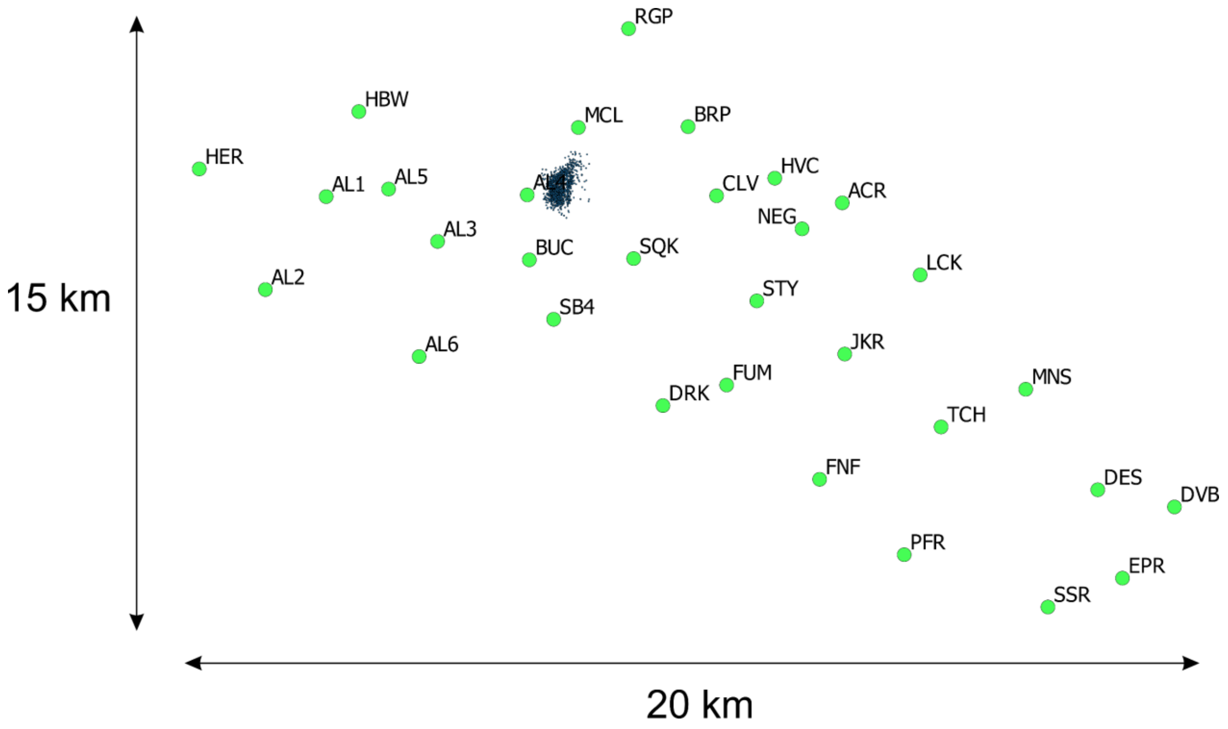

Supplementary Figure S1. Stations of seismic network used for double-difference relocation of ME (green circles). Analyzed seismicity is indicated with small black dots.

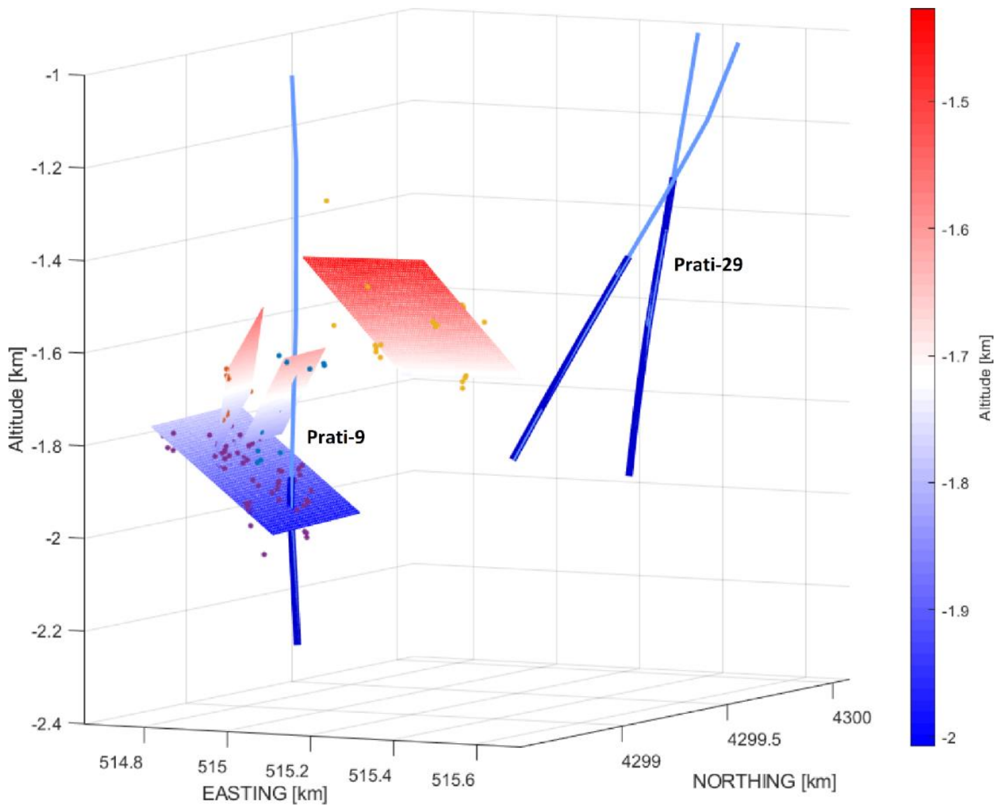

Supplementary Figure S2. Orientation of planes best fitted to ME from groups: A (blue dots), B (orange dots), C (yellow dots) and D (violet dots). Trajectories of injection wells are plotted with blue, open-hole sections with dark blue. The coloring of planes reflects the altitude.

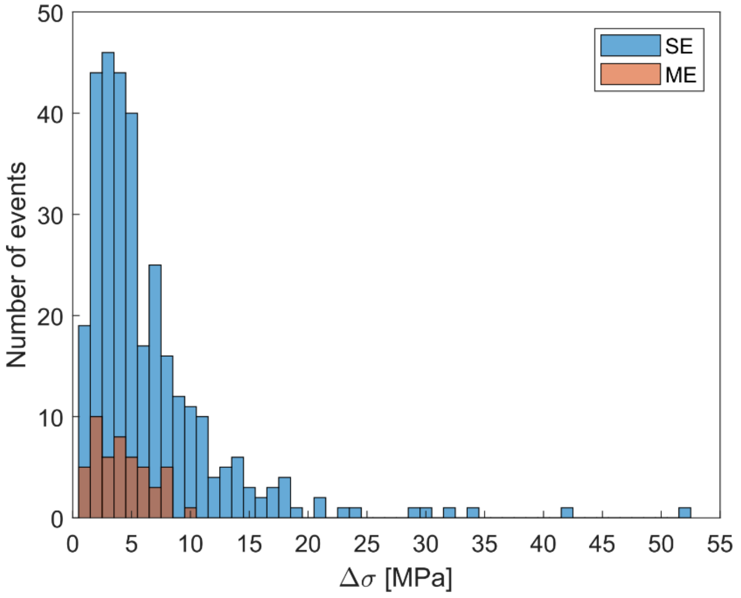

Supplementary Figure S3. Histograms of *Δσ* values of SE and ME.

Tables

Supplementary Table S1. Parameters describing seismic moment release by ME within structures A-D.

Structure	Average seismic moment release [Nm/ME]	Average seismic moment rate [Nm/days]	Total seismic moment released [Nm]	Percentage of total seismic moment released by all ME [%]
A	$3.43 \cdot 10^{11}$	$4.28 \cdot 10^{9}$	$7.55 \cdot 10^{12}$	11.1
B	$1.41 \cdot 10^{11}$	$7.78 \cdot 10^{8}$	$9.85 \cdot 10^{11}$	1.5
C	$4.76 \cdot 10^{11}$	$1.05 \cdot 10^{10}$	$1.33 \cdot 10^{13}$	19.6
**D**	$\mathbf{4.84 \cdot 10^{11}}$	$\mathbf{2.67 \cdot 10^{10}}$	$\mathbf{4.60 \cdot 10^{13}}$	**67.8**
A+B+C+D	x	x	$6.79 \cdot 10^{13}$	100

Supplementary Table S2. Results of statistical comparison of $\Delta\sigma$ distributions with Wilcoxon rank sum test. The null hypothesis stated that $\log(\Delta\sigma)$ of events from Group 1 and Group 2 come from continuous distributions with equal medians.

Group 1	Group 2	Null hypothesis rejection (1 - rejected, 0 - not rejected)	*p*-value	Number of trials with null hypothesis rejected	Number of trials with null hypothesis not rejected
SE	ME	1	$2.6e \cdot 10^{-3}$	8,984	1,016
**SE**	**ME(D)**	**1**	$\mathbf{4.13 \cdot 10^{-4}}$	**9,877**	**123**
SE	ME(A-C)	0	0.52	45	9,955
ME(D)	ME(A-C)	1	0.05	3,156	6,844
ME(D)	ME(C)	1	0.02	5,102	4,898
ME(OL < 20)	ME(OL ≥ 20)	0	0.62	19	9,981

Estimation of relocation errors of ME

Error ellipses (Easting-Northing plane) and depth errors of double-difference relocated earthquakes has been estimated using statistical resampling approach - bootstrap method, as proposed by Waldhauser and Ellsworth [3]. In this approach, we perturb differential times between event pairs with residuals drawn randomly from the distribution of residuals. The procedure is done separately for catalog and cross-correlation data. Then, all the events are relocated with these perturbed differential times. We repeat this procedure 500 times and obtained 500 alternative locations for each event. Final error ellipses contain 95% of obtained bootstrap locations. Error in depth is estimated by taking 95% of obtained event depths. Error ellipses and depth errors are presented below in Supplementary Figures S4-S5 in EN, EZ and NZ sections, respectively. Semi-major, semi-minor axes and azimuth distributions of ellipses within each structure are presented in Supplementary Figures S6-S8. Error in depth is presented in Supplementary Figure S9.

The location errors are generally much higher in N-S direction than E-W. It can be considered as a direct result of seismic network configuration (Supplementary Figure S1). The median length of semi-major axis of error ellipse for ME(A,B,D) equals ca 30 m. The median length of its semi-minor axis for ME(A,B,D) is below 10 m, whereas location uncertainty of these ME in depth equals ca 52 m (median depth error ca 26 m). Structure C is characterized by the highest median errors due to two ME with exceptionally high location errors. Uncertainties of remaining 26 ME(C) are comparable with ME from other structures. We conclude that identified structures A-D can be considered as separate fractures.

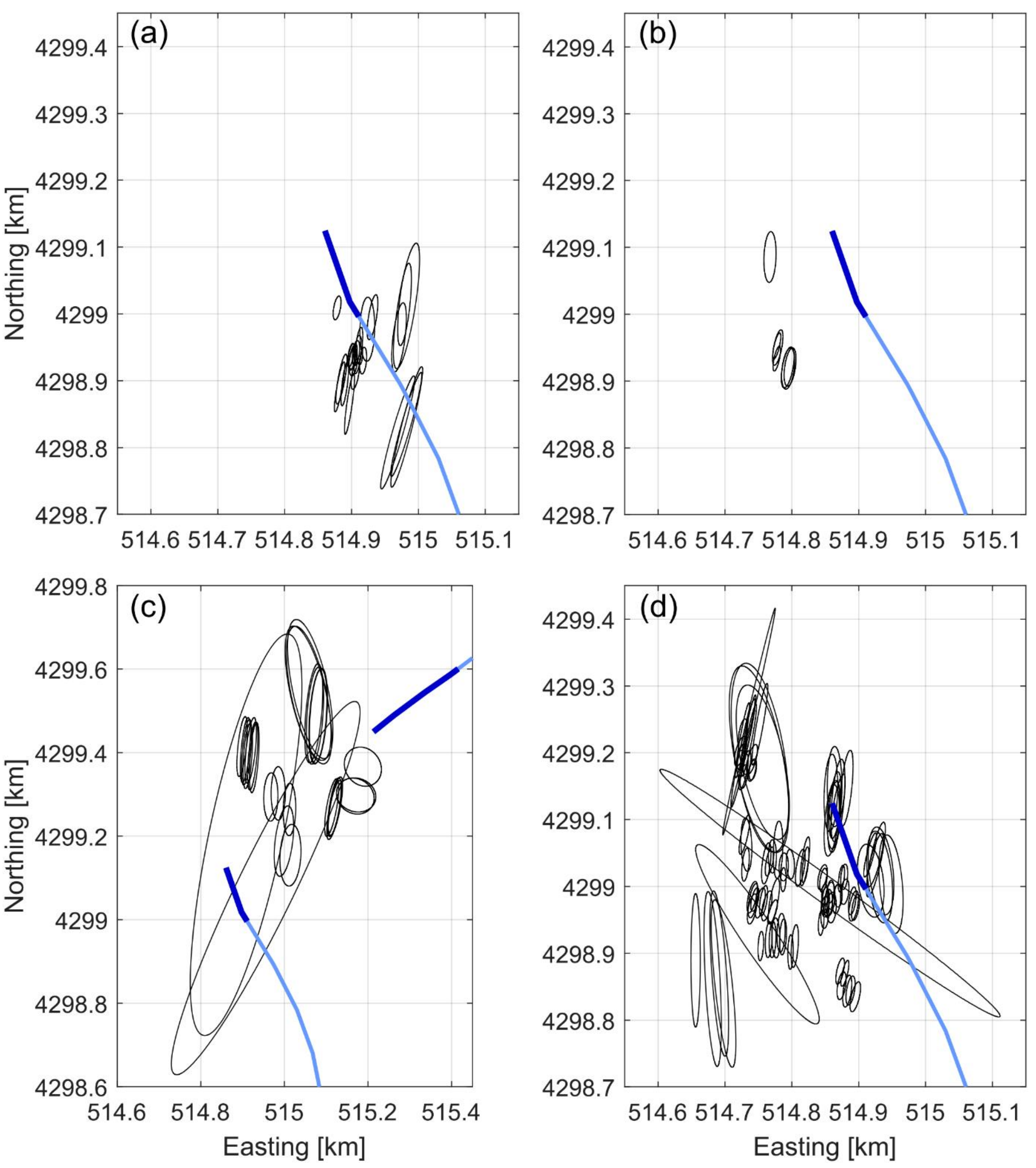

Supplementary Figure S4. Error ellipses presented in Easting-Northing plane. ME from structures A-D are presented in subfigures a-d, accordingly.

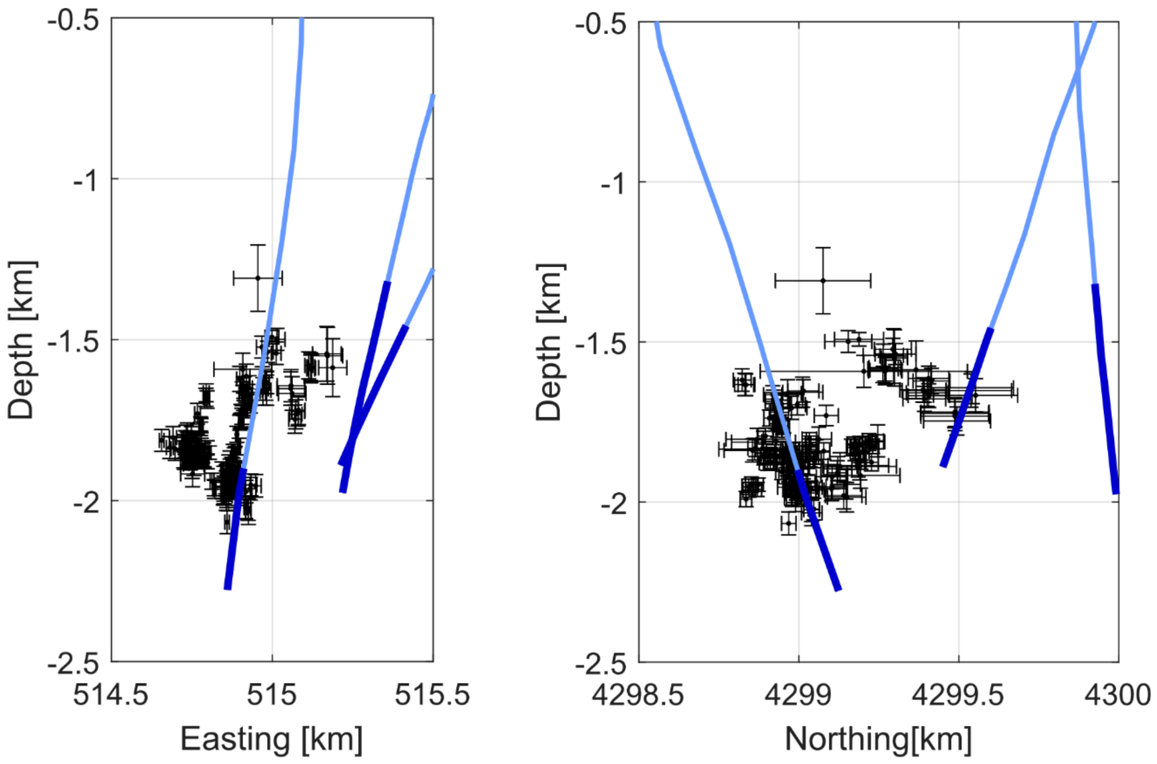

Supplementary Figure S5. Location errors presented in (a) Easting-Depth and (b) Northing-Depth planes.

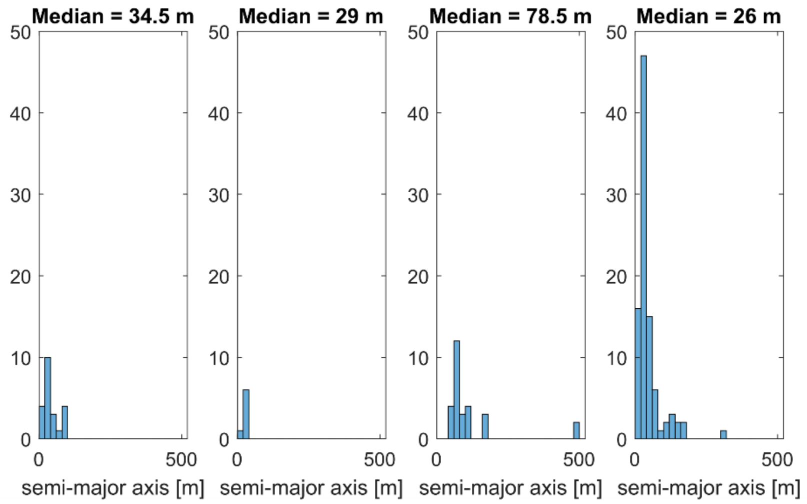

Supplementary Figure S6. Semi-major axes length of error ellipses for ME from structures A-D (from the left).

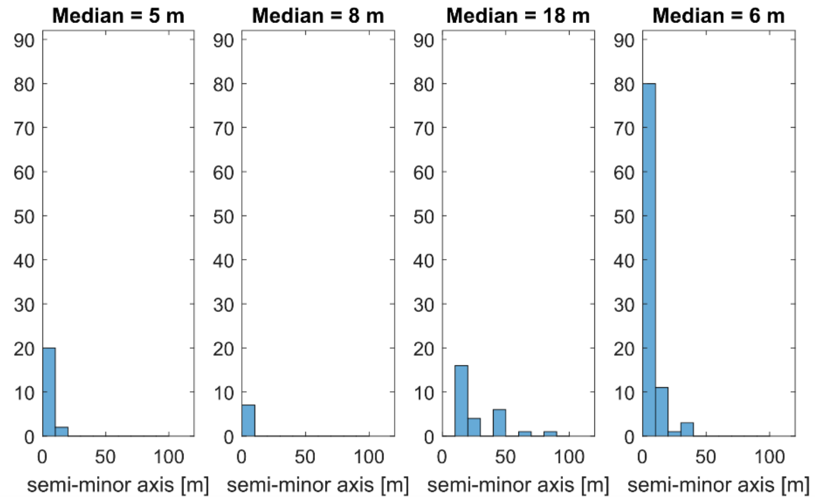

Supplementary Figure S7. Semi-minor axes length of error ellipses for ME from structures A-D (from the left).

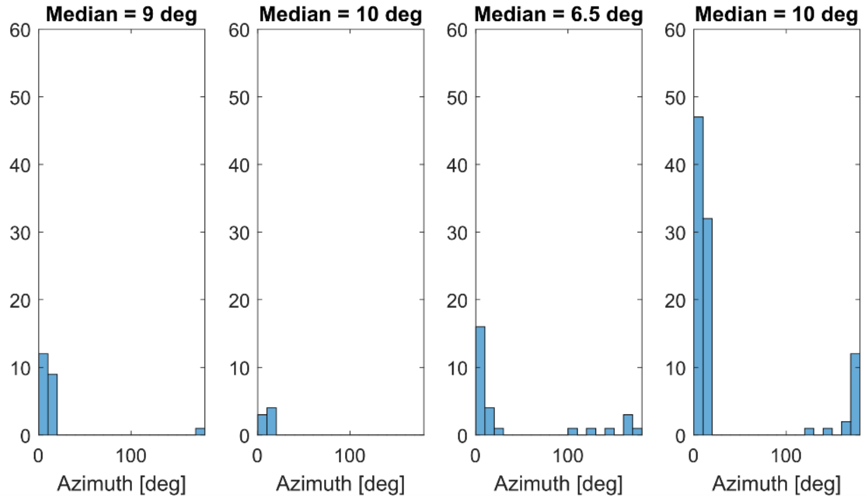

Supplementary Figure S8. Azimuth of error ellipses for ME from structures A-D (from the left).

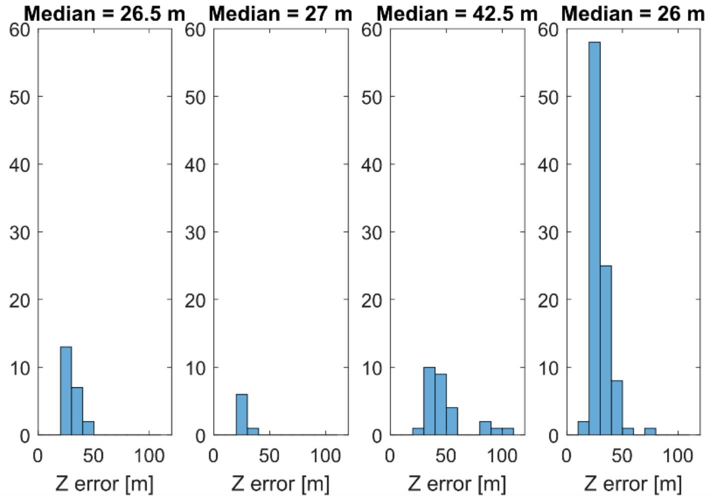

Supplementary Figure S9. Location error in depth for ME from structures A-D (from the left).
